# Retinal Microcirculation as a Correlate of a Systemic Capillary Impairment After Severe Acute Respiratory Syndrome Coronavirus 2 Infection

**DOI:** 10.3389/fmed.2021.676554

**Published:** 2021-07-09

**Authors:** Bettina Hohberger, Marion Ganslmayer, Marianna Lucio, Friedrich Kruse, Jakob Hoffmanns, Michael Moritz, Lennart Rogge, Felix Heltmann, Charlotte Szewczykowski, Julia Fürst, Maximilian Raftis, Antonio Bergua, Matthias Zenkel, Andreas Gießl, Ursula Schlötzer-Schrehardt, Paul Lehmann, Richard Strauß, Christian Mardin, Martin Herrmann

**Affiliations:** ^1^Department of Ophthalmology, University of Erlangen-Nürnberg, Friedrich-Alexander-University of Erlangen-Nürnberg, Erlangen, Germany; ^2^Department of Internal Medicine 1, University of Erlangen-Nürnberg, Friedrich-Alexander-University of Erlangen-Nürnberg, Erlangen, Germany; ^3^Research Unit Analytical BioGeoChemistry, Helmholtz Zentrum München-German Research Center for Environmental Health, Neuherberg, Germany; ^4^Department of Internal Medicine 3, University of Erlangen-Nürnberg, Friedrich-Alexander-University of Erlangen-Nürnberg, Erlangen, Germany

**Keywords:** OCT-angiography, COVID-19, SARS-CoV-2, retina, macula, optic nerve head, microcirculation

## Abstract

Severe acute respiratory syndrome coronavirus 2 (SARS-CoV-2), which causes coronavirus disease 2019 (COVID-19), affects the pulmonary systems via angiotensin-converting enzyme-2 (ACE-2) receptor, being an entry to systemic infection. As COVID-19 disease features ACE-2 deficiency, a link to microcirculation is proposed. Optical coherence tomography angiography (OCT-A) enables non-invasive analysis of retinal microvasculature. Thus, an impaired systemic microcirculation might be mapped on retinal capillary system. As recent OCT-A studies, analyzing microcirculation in two subdivided layers, yielded contrary results, an increased subdivision of retinal microvasculature might offer an even more fine analysis. The aim of the study was to investigate retinal microcirculation by OCT-A after COVID-19 infection in three subdivided layers (I). In addition, short-term retinal affections were monitored during COVID-19 disease (II). Considering (I), a prospective study (33 patients_post−COVID_ and 28 controls) was done. Macula and peripapillary vessel density (VD) were scanned with the Spectralis II. Macula VD was measured in three layers: superficial vascular plexus (SVP), intermediate capillary plexus (ICP), and deep capillary plexus (DCP). Analysis was done by the EA-Tool, including an Anatomical Positioning System and an analysis of peripapillary VD by implementing Bruch's membrane opening (BMO) landmarks. Overall, circular (c_1_, c_2_, and c_3_) and sectorial VD (s_1_-s_12_) was analyzed. Considering (II), in a retrospective study, 29 patients with severe complications of COVID-19 infection, hospitalized at the intensive care unit, were monitored for retinal findings at bedside during hospitalization. (I) Overall (*p* = 0.0133) and circular (c_1_, *p* = 0.00257; c_2_, *p* = 0.0067; and c_3_, *p* = 0.0345). VD of the ICP was significantly reduced between patients_post−COVID_ and controls, respectively. Overall (*p* = 0.0179) and circular (c_1_, *p* = 0.0189) peripapillary VD was significantly reduced between both groups. Subgroup analysis of hospitalized vs. non-hospitalized patients_post−COVID_ yielded a significantly reduced VD of adjacent layers (DCP and SVP) with increased severity of COVID-19 disease. Clinical severity parameters showed a negative correlation with VD (ICP) and peripapillary VD. (II) Funduscopy yielded retinal hemorrhages and cotton wool spots in 17% of patients during SARS-CoV-2 infection. As VD of the ICP and peripapillary regions was significantly reduced after COVID-19 disease and showed a link to clinical severity markers, we assume that the severity of capillary impairment after COVID-19 infection is mapped on retinal microcirculation, visualized by non-invasive OCT-A.

## Introduction

In December 2019, several cases of a severe and unknown pneumonia were diagnosed in Wuhan (1). Severe acute respiratory syndrome coronavirus 2 (SARS-CoV-2) was identified as a disease causative agent, and clinical features were summarized as coronavirus disease 2019 (COVID-19). Ranking to a worldwide healthcare problem, COVID-19 pandemic was declared on March 11, 2020. On the molecular basis, SARS-CoV-2 enters human cells via the angiotensin-converting enzyme-2 (ACE-2) receptor (2, 3). After binding via receptor-binding domain of the spike protein, a target cell protease splits the spike, enabling virus entry mediated by the spike fusion peptide (4–6). The natural target of the ACE-2 receptor, ACE-2, was first discovered in 2000 (7). ACE-2, part of the renin–angiotensin system (RAS), converts angiotensin II (Ang II) to angiotensin (Ang)-1-7. ACE-2 and Ang-1-7 are assumed to prevent atherosclerosis and protect endothelial cells via inhibition of inflammation (8). The latter one was seen to reduce oxidative stress via the MAS receptor (9, 10). Several studies have suggested a link of ACE-2 to vascular diseases (11, 12). Considering this, ACE-2 deficiency consecutively was observed to cause vascular inflammation and atherosclerosis (8, 13). In addition, e.g., expression of adhesion molecules (e.g., VCAM), monocyte chemoattractant protein-1 (MCP-1), and interleukin 6 (IL-6) were significantly increased (8, 14, 15). It is hypothesized that SARS-CoV-2 targeting the ACE-2 receptor features molecular characteristics of ACE-2 deficiency (16, 17).

Data on ACE-2 and its receptor in human retina are rare in literature up to now. The ACE-2 protein was detected in human retina by Western blotting analysis (18). Structural analysis in animal models (rodent and porcine) indicated that ACE-2 is present in the inner granular and nuclear layers (19, 20). Thus, it is assumed that involvement of SARS-CoV-2 might also be present in the retina itself. If we assume that SARS-CoV-2 infection might mimic an ACE-2 deficiency, we hypothesize that retinal microcirculation might be affected, especially, in the inner granular and nuclear layers. In addition, during COVID-19, the circulating neutrophils are activated, and the population of low-density granulocytes is highly increased. These cells are prone to form neutrophil extracellular traps (NETs). The latter have been shown to occlude pulmonary vessels in active COVID-19, which is referred to as immunothrombosis. Hepatic and glomerular vessels were also affected (21–23). We hypothesized that this kind of vasculopathy may also affect retinal microvasculature.

**Figure 1:**
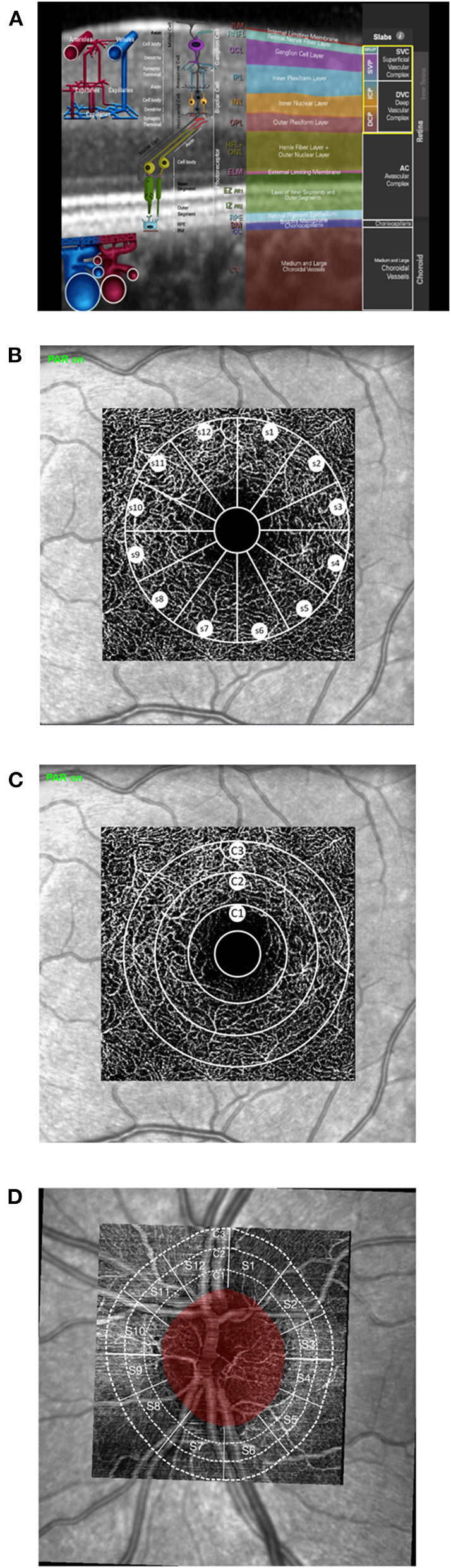
Morphometric and quantitative analyses of optical coherence tomography angiography (OCT-A): **(A)** anatomic correlation of retinal layers to superficial capillary plexus (SVP), intermediate capillary plexus (ICP), and deep capillary plexus (DCP; image courtesy by Heidelberg Engineering, Heidelberg, Germany). **(B–D)** Schematic sketch of quantitative OCT-A analysis of the macula **(B,C)** and peripapillary scans **(D)** with the Erlangen Angio Tool: circular (**B**; c_1_, c_2_, and c_3_) and sectorial VD (**C**; 12 sectors, s_1_-s_12_; à 30°).

Optical coherence tomography angiography (OCT-A) is a non-invasive technique, visualizing retinal microcirculation. Several devices can be used right now, most of them analyzing vessel density (VD) and characteristics of microcirculation in two different retinochoroidal layers (superior vs. deep). Up to now, there is only one device available (Spectralis II; Heidelberg Engineering, Heidelberg, Germany) enabling analysis of retinal microcirculation in three layers with high resolution: superficial vascular plexus (SVP), intermediate capillary plexus (ICP), and deep capillary plexus (DCP). Those three layers correlate well with human anatomy (24). The SVP correlates with the ganglion cell layer (GCL) and part of the inner plexiform layer (IPL), the ICP with part of the IPL and inner nuclear layer (INL), and the DCP with part of the INL and outer plexiform layer (OPL), respectively (Figure 1A). This more fine resolution of OCT-A data enables an even more detailed analysis of retinal microcirculation. Recent studies investigated changes in retinal microcirculation early after COVID-19 infection (2 weeks until 1 month) by OCT-A devices, subdividing the OCT-A scan into two layers (superficial retinal capillary plexus and deep retinal capillary plexus) (25, 26). Thus, it was the aim of the present study to investigate retinal microvasculature of the macula, subdivided into three retinal layers, and peripapillary region in patients after long-term COVID-19 infection as compared with control eyes. In addition, short-term alterations of retinal findings and clinical outcome were monitored in patients with severe COVID-19 complications.

## Methods

### Subjects

#### Long-Term Effect of Coronavirus Disease 2019 Infection on Retinal Microcirculation

A prospective study was done analyzing long-term effects of COVID-19 infection on retinal microcirculation: 61 eyes of 61 persons were included: 33 eyes of patients_post−COVID_ (13 females and 20 males) and 28 control eyes (20 females and 8 males). Infection of COVID-19 was confirmed by a positive result of real-time, reverse transcription–polymerase chain reaction (PCR). Patients_post−COVID_ were recruited after hospitalization in the Department of Internal Medicine 1, University of Erlangen-Nürnberg, and from local residents, which were not hospitalized for COVID-19 infection. Time after positive SARS-CoV-2 PCR test was 138.13 ± 70.67 days (range 34–281 days). All eyes had no history of a previously known retinal or papillary disorder. No ocular laser therapy or surgery has been performed. Control eyes did not show ocular disorders or had a history of laser therapy or ocular surgery. All patients and subjects underwent measurement of best-corrected visual acuity (BCVA) and intraocular pressure (IOP). Axial length was measured by IOLMaster (Zeiss, Oberkochen, Germany). Demographic data can be seen in Table 1. The study has been approved by the local ethics committee and performed in accordance with the tenets of the Declaration of Helsinki.

**Table 1:** Demographic data of the prospective study cohort for long-term effects of coronavirus disease 2019 (COVID-19) infection: gender, age, best-corrected visual acuity (BCVA), intraocular pressure (IOP), and axial length in patients after COVID-19 infection (patients_post−COVID_) and controls.

	**Gender (f/m)**	**Age [years]**	**BCVA**	**IOP [mmHg]**	**Axial length [mm]**
Patients_post−COVID_	12/20	43.7 ± 19	0.9 ± 0.2	14 ± 3	23.7 ± 1.1
Controls	20/8	29.2 ± 12	1.2 ± 0.2	15 ± 3	23.9 ± 0.8

#### Short-Term Effect of Severe Coronavirus Disease 2019 Infection on Retinal Microcirculation

Short-term effects of severe COVID-19 infection on retinal microcirculation were assessed retrospectively: 29 patients with severe complications of COVID-19 infection, hospitalized at the intensive care unit at the University of Erlangen, were monitored by an ophthalmologist for retinal findings. Mean age was 60.9 ± 15 years. Infection of SARS-CoV-2 was confirmed by a positive result of real-time, reverse transcription-PCR. Funduscopy was done at bedside during hospitalization.

### Optical Coherence Tomography Angiography and Erlangen-Angio-Tool (Version 3.0)

Macula and peripapillary VD was scanned with the Heidelberg Spectralis II (Heidelberg, Germany). Macula VD was measured in three microvascular layers: SVP (thickness: 80 μm), ICP (thickness: 50 μm), and DCP (thickness: 40 μm). The scans were based on an angle of 15° × 15° and the highest commercially available lateral resolution of 5.7 μm/pixel. Scan size was 2.9 × 2.9 mm (total scan size 8.41 mm^2^; diameter of inner ring: 0.8 mm; diameter of outer ring: 2.9 mm). All scans were analyzed by the EA-Tool (version 3.0), which was coded in Matlab (The MathWorks, Inc., Natick, USA, R2017b). This software tool enables quantification of macula and peripapillary VD with high reliability and reproducibility (27).

EA-Tool version 3.0 is an advanced quantification software, including an Anatomical Positioning System (APS; part of Glaucoma Module Premium Edition [GMPE], Heidelberg Engineering, Heidelberg, Germany) allowing alignment of OCT-A scans to each patient's individual FoBMOC (Fovea-to-Bruch's Membrane Opening-Center) axis, during scan acquisition or retrospectively (“APS-ify”). In addition, peripapillary VD can be analyzed by implementing the BMO landmarks (BMO-based peripapillary VD). APS and BMO coordinates were exported by SP-X1902 software (prototype software, Heidelberg Engineering, Heidelberg, Germany) (28). After manual checking for correct segmentation and artifacts, the analysis was performed. Overall, circular (c_1_, c_2_, and c_3_) and sectorial VD (12 sectors, s_1_-s_12_; à 30°) of the macula and peripapillary scans were analyzed (Figure 1). In addition, analysis of the foveal avascular zone (FAZ) was done.

### Statistical Analysis

For the variable VD of the SVP, ICP, and DCP, we applied a mixed model analysis with sectors as repetition measures. The variables gender and age were introduced in the model as covariates. The model has a random intercept and 12 time measurements defined as repetitions. The interactions between diagnosis and sectors were also calculated together with the *p*-values of the multiple comparisons (after the Tukey–Kramer adjustment). The 95% CI was reported together with the *p*-values. As the experimental design was unbalanced, we estimated the least squares (LS)-means that correspond to the specified effects for the linear predictor part of the model, and the relative confidence limits. LS-means are closer to reality and represent even more real data, when cofactors occur, compared with means. Moreover, we calculated the within-subjects effects using the interactions between the variables (patients_post−COVID_ and controls) ^*^ sectors.

For the variables c_1_, c_2_, and c_3_ of the SVP, ICP, and DCP, we applied a covariance analysis (where gender and age were set in the model as covariates). The diagnosis was set as a class variable with two levels (patients_post−COVID_ and controls). Type III SS test of the multiple comparisons (adjusted with Tukey–Kramer) and 95% CI were reported to evaluate the contribution of the factor. The LS-means were calculated. All the statistical elaborations were done using SAS version 9.3 (SAS Institute Inc., Cary, NC, USA).

## Results

### Long-Term Effect of Coronavirus Disease 2019 Infection on Retinal Macula Microcirculation

The covariance models were done with age and gender as covariates. The *p*-values were adjusted with Tukey's test. In addition, a mixed model with the sector's variables as repeated measures, age and gender as covariates, and patients' group as variables (patients_post−COVID_ vs. controls) was performed.

LS-mean macula VD was 30.25 ± 0.5 (SVP), 22.74 ± 0.5 (ICP), and 24.02 ± 0.6 (DCP) in controls. LS-mean macula VD was 29.51 ± 0.5 (SVP), 21.02 ± 0.4 (ICP), and 23.08 ± 0.5 (DCP) in patients_post−COVID_. Mixed model analysis yielded a significant age effect on macula VD in the SVP (*p* = 0.0015), ICP (*p* = 0.0002), and DCP (*p* = 0.0028). After age correction of the VD data, additional regional variations (i.e., sectorial effect) of macula VD were observed in the SVP (*p* < 0.0001), ICP (*p* < 0.0001), and DCP (*p* < 0.0001). Gender did not affect macula VD in all three microvascular layers (*p* > 0.05).

After age correction of VD data, overall macula VD was significantly reduced in the ICP between patients_post−COVID_ and controls (*p* = 0.0133). Yet overall macula VD of the SVP and DCP was not significantly different between patients_post−COVID_ and controls (*p* > 0.05), respectively.

**Figure 2:**
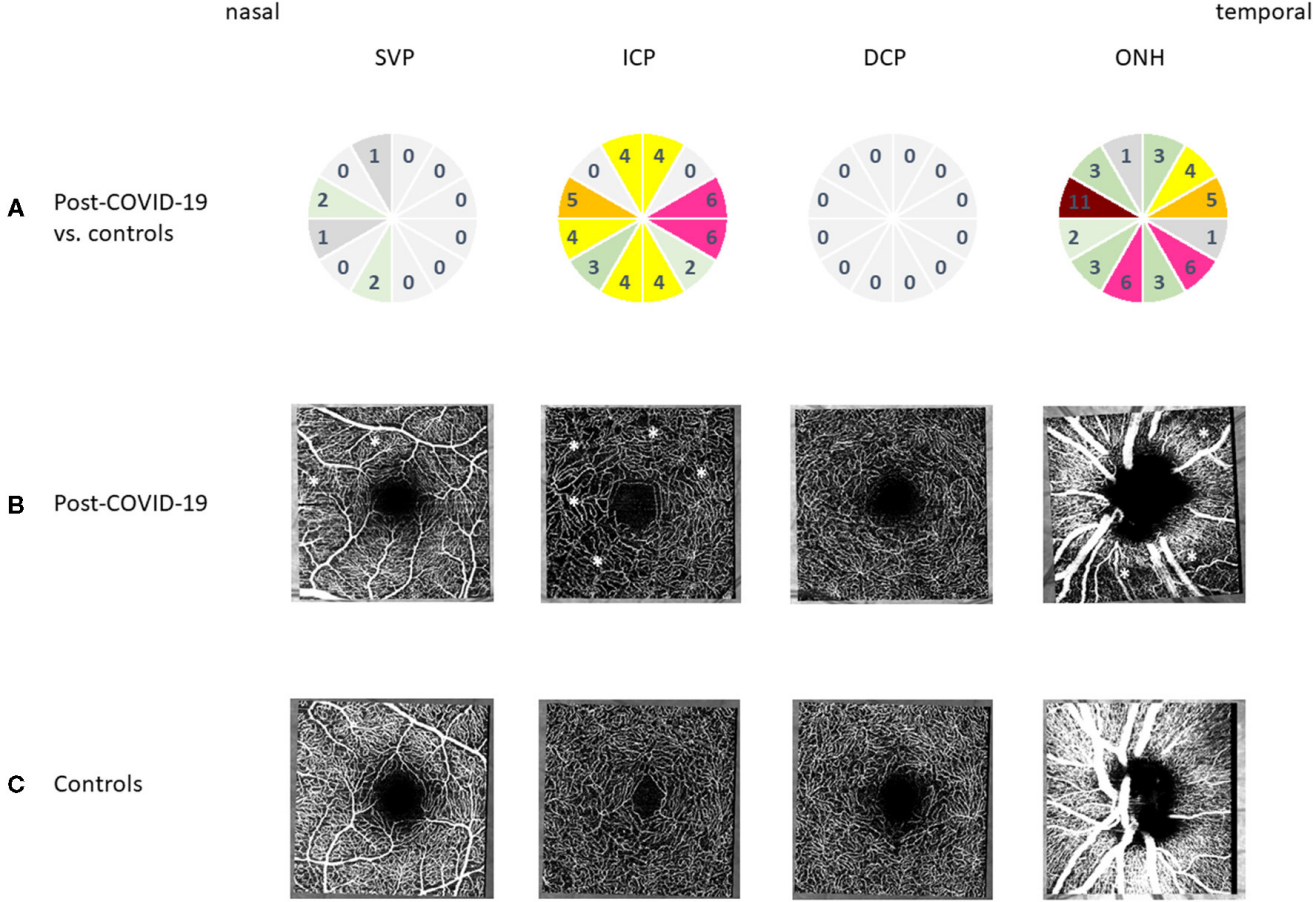
Microcirculation of the macula and peripapillary regions after coronavirus disease 2019 (COVID-19) infection compared to controls: **(A)** qualitative analysis of the number of significant interactions between vessel density of each sector (s_1_-s_12_) of macula optical coherence tomography angiography (OCT-A) in superficial vascular plexus (SVP), intermediate capillary plexus (ICP), and deep capillary plexus (DCP) and optic nerve head (ONH) by color coding (red, *n* ≥ 8; pink, *n* = 6–7; orange, *n* = 5; yellow, *n* = 4; green, *n* = 2–3; gray, *n* = 0–1) between controls and post-COVID-19 eyes. **(B)** Representative OCT-A scans of the macula (SVP, ICP, and DCP) and peripapillary regions after COVID-19 infection (^⋆^Areas of reduced vessel density). **(C)** Representative OCT-A scans of the macula (SVP, ICP, and DCP) and peripapillary region in controls.

After age correction of VD data, subgroup analysis of the three peri-macula circles (c_1_, c_2_, and c_3_) yielded a significant reduction of VD of c_1_, c_2_, and c_3_ of the ICP in patients_post−covid_ compared with controls (*p* = 0.00257; *p* = 0.0067; and *p* = 0.0345). No significant differences of macula VD were observed in c_1_, c_2_, and c_3_ of the SVP (*p* > 0.05) and DCP (*p* > 0.05). In addition to the analysis of peri-macula VD, a sectorial analysis was done, showing significant interactions between patients_post−COVID_ and controls in the ICP (Figure 2). Color-coded numbers of all significant interactions between each sector are shown in Figure 2 for the SVP, ICP, and DCP (number of significant interactions: red, *n* ≥ 8; pink, *n* = 6–7; orange, *n* = 5; yellow, *n* = 4; green, *n* = 2–3; and gray, *n* = 0–1).

### Long-Term Effect of Coronavirus Disease 2019 Infection on Retinal Peripapillary Microcirculation

Peripapillary LS-mean VD was 42.12 ± 0.8 (controls) and 39.37 ± 0.7 (patients_post−COVID_). Type 3 tests of fixed effects showed a significant influence of age (*p* = 0.0013), yet no gender effect on peripapillary VD (*p* > 0.05). After age correction of VD data, an additional sectorial effect was observed (*p* < 0.0001). After age correction of VD data, overall peripapillary VD was significantly reduced between patients_post−COVID_ and controls (*p* = 0.0179). In addition, c_1_ was significantly lowered in patients_post−COVID_ compared with controls (*p* = 0.0189), yet not c_2_ and c_3_ (*p* > 0.05). Considering the sectorial effect with localized and fine alterations of microcirculation, a distinct analysis of each single sector (s_1_-s_12_) was added. Figure 2 shows a color-coded number of significant interactions between each hour in patients_post−COVID_ and controls (number of significant interactions: red, *n* ≥ 8; pink, *n* = 6–7; orange, *n* = 5; yellow, *n* = 4; green, *n* = 2–3; gray, *n* = 0–1).

### Long-Term Effect of Coronavirus Disease 2019 Infection on Foveal Avascular Zone Characteristics

FAZ was 0.28 ± 0.02 (SVP), 0.19 ± 0.02 (ICP), and 0.27 ± 0.07 (DCP) in controls. Patients_post−COVID_ showed a FAZ of 0.24 ± 0.02 (SVP), 0.16 ± 0.015 (ICP), and 0.35 ± 0.06 (DCP). Mixed model analysis yielded no age effect on FAZ of the SVP, ICP, and DCP (*p* > 0.05). Gender did affect FAZ in the ICP significantly (*p* = 0.042), yet not in the SVP (*p* > 0.05) and DCP (*p* > 0.05). No significant differences were observed between patients_post−COVID_ and controls for FAZ in all three microvascular layers (*p* > 0.05).

### Analysis of Long-Term Effect of Coronavirus Disease 2019 Infection Between Hospitalized and Non-hospitalized Patients

We applied the same model (mixed model) to find differences in the SVP, ICP, DCP, and peripapillary region between the patients_post−COVID_ being hospitalized and non-hospitalized during SARS-CoV-2 infection. These groups showed overall LS-mean of 30.00 ± 0.9 (non-hospitalized) and 28.12 ± 0.9 (hospitalized) in the SVP, 21.43 ± 0.8 (non-hospitalized) and 19.58 ± 0.8 (hospitalized) in the ICP, and 24.23 ± 0.9 (non-hospitalized) and 21.00 ± 0.9 (hospitalized) in the DCP. Peripapillary LS-mean VD was 39.51 ± 1.3 (non-hospitalized) and 37.84 ± 1.3 (hospitalized).

A significantly reduced overall LS-mean VD was observed in the DCP of hospitalized patients_post−COVID_ compared with non-hospitalized ones (*p* = 0.0304, Table 2A). Contrarily, overall LS-mean of the SVP and ICP was similar between both groups (*p* > 0.05). LS-mean VD of the SVP, ICP, and DCP of patients_post−COVID_ who were hospitalized and non-hospitalized during SARS-CoV-2 infection can be seen in Table 3. Considering fine variations of VD, a circular analysis was done: VD of c_2_ (*p* = 0.0468) and c_3_ of the DCP (*p* = 0.0232) and VD of c_1_ of the SVP (*p* = 0.0465) were significantly reduced in patients_post−COVID_ being hospitalized vs. non-hospitalized during SARS-CoV-2 infection. Comparing this two subgroups of patients_post−COVID_ with controls, VD of c_3_ of the DCP was significantly reduced in hospitalized < non-hospitalized < controls (*p* = 0.015), yet not c_2_ (DCP) and c_1_ (SVP, *p* > 0.05). The comparison of each group with its respective *p*-value can be seen in Table 2B. In addition, no differences were observed for c_1_ (DCP); c_2_ and c_3_ (SVP); c_1_, c_2_, and c_3_ (ICP); and c_1_, c_2_, and c_3_ (peripapillary) of hospitalized vs. non-hospitalized patients_post−COVID_ (*p* > 0.05).

**Table 2:** Overall (A) and c_3_ of (B) vessel density of deep capillary plexus (DCP) of patients_post−COVID_ who were hospitalized and non-hospitalized during COVID-19 infection: (A) mixed model analysis showed a significantly reduced overall LS-mean vessel density (VD) for hospitalized (coded as 1) compared with non-hospitalized patients (coded as 0); (B) general linear model (non-hospitalized, hospitalized patients_post−COVID_ and controls) showed a significantly reduced VD of c_3_ of DCP for hospitalized < non-hospitalized < controls.

**(A)**													
**Type 3 tests of fixed effects of vessel density of DCP**													
**Effect**	**Num DF**	**Den DF**	**F value**	**Pr** **>** **F**									
Hospitalized	1	29	5.18	0.0304									
Gender	1	29	2.58	0.1188									
Age	1	29	0.06	0.8099									
Sector	11	352	2.92	0.001									
**Least squares means of vessel density of DCP**													
**Effect**	**Groups**	**Estimate**	**Standard error**	**DF**	***t***	**Pr** **>** **|t|**	**Alpha**	**Lower**	**Upper**				
Hospitalized	**0**	24.23	0.8928	29	27.14	<0.0001	0.05	22.404	26.06				
Hospitalized	**1**	21.003	0.9006	29	23.32	<0.0001	0.05	19.161	22.85				
**Differences of least squares means of vessel density of DCP**													
**Effect**	**Group**	**Group**	**Estimate**	**Standard Err**.	**DF**	***t*** **Value**	**Pr** **>** **|t|**	**Adj p**	**Alpha**	**Lower**	**Upper**	**Adj lower**	**Adj Upper**
Hospitalized	**0**	**1**	3.23	1.42	29	2.28	0.03	0.03	0.05	0.33	6.13	0.33	6.13
**(B)**													
**Least squares means of c**_**3**_ **of DCP for effect groups**													
**t for H0: LS-mean(i)** **=** **LS-mean(j)/Pr** **>** **|t|**													
**Dependent Variable: c**_**3**_ **of DCP**													
**i/j**	**Non-hospitalized**	**Hospitalized**	**Control**										
Non-hospitalized		2.822824	0.124304										
		0.0178	0.9915										
Hospitalized	−2.82282		−2.72584										
	0.0178		0.0229										
Control	−0.1243	2.725835											
	0.9915	0.0229											

**Table 3:** Long-term effect of COVID-19 infection on circular vessel density (c_1_, c_2_, and c_3_) of SVP, ICP, DCP, and peripapillary region between hospitalized (coded as 1) and non-hospitalized (coded as 0) patients: LS-mean, 95% confidence limits.

		**Groups**	**LS-mean**	**95% confidence limits**	
SVP	c_1_	0	25.50	23.23	27.76
		1	21.84	19.55	24.12
	c_2_	0	30.58	28.68	32.48
		1	29.03	27.11	30.95
	c_3_	0	32.01	30.10	33.91
		1	30.39	28.47	32.31
ICP	c_1_	0	19.25	17.60	20.91
		1	18.32	16.65	19.99
	c_2_	0	21.40	19.79	23.01
		1	19.84	18.21	21.46
	c_3_	0	22.60	20.85	24.36
		1	19.98	18.21	21.75
DCP	c_1_	0	19.76	17.64	21.88
		1	16.65	14.51	18.79
	c_2_	0	24.86	23.17	26.54
		1	22.14	20.44	23.84
	c_3_	0	26.00	23.95	28.05
		1	22.18	20.11	24.25
Peripapillary	c_1_	0	40.36	37.14	43.58
		1	39.74	36.50	42.99
	c_2_	0	38.85	35.19	42.51
		1	37.43	33.74	41.13
	c_3_	0	30.46	25.86	35.07
		1	33.35	28.70	38.00

*COVID-19, coronavirus disease 2019; SVP, superficial vascular plexus; ICP, intermediate capillary plexus; DCP, deep capillary plexus*.

### Correlation of Clinical Data During Hospitalization and Optical Coherence Tomography Angiography Parameters

Clinical characteristics of the hospitalized patients_post−COVID_ (*n* = 17) during their hospitalization can be seen in Table 4. The non-hospitalized patients_post−COVID_ (*n* = 16; six females and 10 males) were at home during their SARS-CoV-2 infection without the necessity of being inpatients. Only few preexisting conditions were monitored in the non-hospitalized group: asthma (1/16, 6%) and status after cardiac ablation (1/16, 16%). The correlation of VD of each microvascular layer (SVP, ICP, and DCP) with clinical parameters during hospitalization can be seen in Table 5. Interestingly, negative correlations were observed for the highest level of D-dimer and the highest level of Glutamat-Pyruvat-Transaminase (GPT) with peripapillary VD (circular analysis, c_1_ and c_2_). In addition, stage at diagnosis correlated negatively with VD in the ICP (overall, c_1_-c_3_), peripapillary region (overall, c_1_-c_3_), and SVP (overall, c_2_ and c_3_). Thus, the worse the COVID-19 infection had been, the more reduced the VD was measured in OCT-A.

**Table 4:** Clinical data of hospitalized patients with coronavirus disease 2019 (COVID-19) infection (*n* = 17): preexisting condition, immunosuppressive medication (past 3 months), smoker, systemic therapy, thrombosis prophylaxis, clinical follow-up during hospitalization at the intensive care unit, and body mass index (BMI) (*n* = 13).

**Clinical characteristics**		
	**11 males (65%)**	**6 females (35%)**
Stage at diagnosis	Non-severe	14 (82%)
	Severe	2 (12%)
	Critical	1 (6%)
Median hospital stay	8 days (range 3–46 days)	
Preexisting condition	Chronic heart failure	1 (6%)
	Peripheral artery occlusive disease	1 (6%)
	Hypertension	4 (24%)
	Diabetes	1 (6%)
	Chronic kidney disease	1 (6%)
	Rheumatic disorder	1 (6%)
	Atrial fibrillation	1 (6%)
	Immunosuppression	3 (18%)
	Chemotherapy	2 (12%)
COVID treatment	Hydroxychloroquine	12 (63%)
	Steroids	1 (6%)
	Reconvalescent plasma	1 (6%)
Thrombosis prophylaxis(e.g., heparin or low-molecular-weight heparin prophylactic dose)		15 (88%)
Heparin therapeutic dose		1 (6%)

*Clinical data were collected using REDCap electronic data capture tools hosted at University Hospital Erlangen (29). Clinical phases were defined according to the definition of stages proposed by the LEOSS registry (30). Severe phase was mainly characterized by need of oxygen supplementation; critical phase was mainly defined by need of mechanical ventilation*.

**Table 5:** Correlation analysis of clinical data during hospitalization and vessel density in SVP, ICP, and DCP: time between positive SARS-CoV-2 test and OCT-A; stage at diagnosis (non-severe, severe, and critical) and the highest level of D-dimer and Glutamat-Pyruvat-Transaminase (GPT) during hospitalization.

		**Time [positive SARS-CoV-2 test until OCT-A; days]**	**Stage at diagnosis [non-severe (1), severe (2), critical (3)]**	**Highest level of D-dimer [ng/ml] during hospitalization**	**Highest level of GPT[U/L] during hospitalization**
SVP	Overall	0.15	−0.10	0.45	0.26
	c_1_	0.34	0.23	0.51	0.33
	c_2_	0.09	−0.12	0.45	0.24
	c_3_	0.08	−0.21	0.34	0.20
ICP	Overall	0.10	−0.11	0.46	0.14
	c_1_	0.18	−0.02	0.47	0.28
	c_2_	0.11	−0.06	0.51	0.14
	c_3_	0.07	−0.17	0.39	0.08
DCP	Overall	0.12	0.12	0.29	0.03
	c_1_	0.22	0.22	0.34	0.09
	c_2_	0.08	0.08	0.26	0.00
	c_3_	0.11	0.11	0.27	0.03
Peripapillary	Overall	0.26	−0.38	0.09	0.11
	c_1_	0.28	−0.43	−0.21	−0.10
	c_2_	0.01	−0.50	−0.23	−0.13
	c_3_	0.24	−0.28	0.18	0.11

*Severe phase was mainly characterized by need of oxygen supplementation; critical phase was mainly defined by need of mechanical ventilation; Pearson's correlations: negative value represent a worsening of VD with increasing clinical parameter.*

*SVP, superficial vascular plexus; ICP, intermediate capillary plexus; DCP, deep capillary plexus; SARS-CoV-2, severe acute respiratory syndrome coronavirus 2; OCT-A, optical coherence tomography angiography; VD, vessel density*.

### Short-Term Effect of Severe Coronavirus Disease 2019 Infection on Retinal Microcirculation

**Figure 3:**
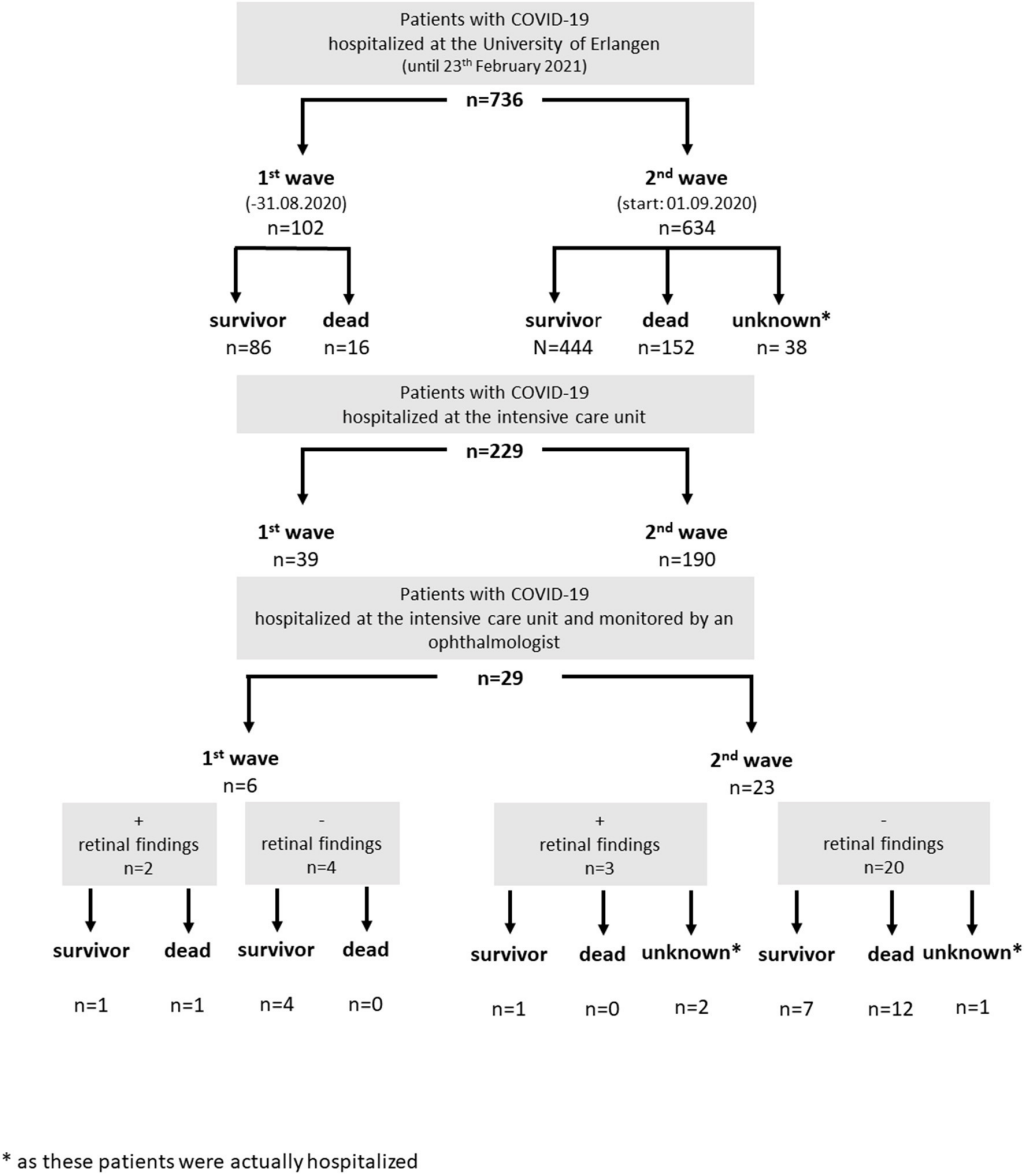
Hospitalized patients with coronavirus disease 2019 (COVID-19) infection at the University of Erlangen (status February 23, 2021): a subgroup of patients of the intensive care unit was monitored by an ophthalmologist for retinal findings (subdivided by their clinical outcome).

Twenty-nine persons with severe complications of COVID-19 infection were monitored for retinal finding during hospitalization in the intensive care unit: six persons (first wave, until August 31, 2020) and 23 persons (second wave). None of them showed an involvement of the cornea, anterior chamber, and vitreous body in terms of infection. Retinal findings (e.g., retinal bleedings and cotton wool spots) were observed in 17% (5/29 persons, Figure 3).

## Discussion

COVID-19 infection had reached to a pandemic healthcare problem during 2020. Next to pulmonary complications, microcirculation (e.g., vasculitis and thromboembolism) is impaired during SARS-CoV-2 infection (16, 31). Endothelial cells, attacked by SARS-CoV-2, were observed to show either inclusion bodies of the endothelial cells or an endotheliitis (32). The inflammation itself or microthrombosis can consecutively be the starting point on an impaired capillary microcirculation and afterwards an endothelial dysfunction (32). An immunological component has been proposed for the microthrombosis (immune thrombosis) (33). The data of the present study emphasized that capillary microcirculation is restricted even in patients after COVID-19 infection. Especially, the retinal microvascular layer (ICP), showing ACE-2 receptor in animal models (inner nuclear and inner plexiform layer), was affected in patients_post−COVID_ and showed significantly reduced VD. The worse the stage at diagnosis was, the worse the VD of the ICP was observed even after a period after COVID-19 infection. In addition, peripapillary VD was significantly reduced after SARS-CoV-2 infection. Considering severity of COVID-19 disease, the more increased the level of D-dimers and GPT were observed during hospitalization, the more reduced the peripapillary VD was measured in OCT-A scans. This effect was emphasized by the finding that patients_post−COVID_ who were hospitalized during COVID-19 infection showed even significantly reduced VD in the adjacent microvascular layers next to the ICP (i.e., SVP, and DCP) than the patients_post−COVID_ who did not need hospitalization during COVID-19 infection, and even when compared with controls (c_3_ of DCP). These results argue for a critical impairment of retinal microcirculation after COVID-19 infection, accented in the ICP, yet affecting additional adjacent microvascular layers after even worse COVID-19 infections.

An ocular involvement of COVID-19 infection has been described for several times. Patients' symptoms vary from normal till blurred vision with accompanying epiphora, discharge, or itching (34–36). Clinical findings can be summarized as follicular conjunctivitis (35), pseudomembranous or hemorrhagic conjunctivitis (37), or keratoconjunctivitis (38). Data of animal models showed that SARS-CoV-2 might affect uveal (e.g., anterior pyogranulomatous uveitis and choroiditis) or neuronal tissue (e.g., retinitis or optic neuritis) (39–43). *In vivo* data of the present study yielded that none of the patients showed signs of uveitis or vasculitis. Yet self-limiting bleedings or cotton wool spots were monitored, being a marker of an impaired retinal microcirculation. This sign of capillary restriction was observed in even 17% of the COVID-19 patients during their hospitalization at the intensive care unit. To the best of our knowledge, the data of the present study show this high percentage of short-term affections of retinal findings during COVID-19 infection for the first time. The only data available in literature up to now offer percentages of 12.9–22% in patients after COVID-19 infection (26, 44).

Yet the percentage of an affected retinal microcirculation seemed to be even higher as OCT-A results suggest. Retinal microcirculation can be monitored by non-invasive OCT-A technology with a high resolution of the scanned structures. There are only two previous clinical studies up to now, investigating microvasculature of the macula with OCT-A after COVID-19 infection, yet showing contrary results. As one study yielded a significant reduction of VD in the superficial capillary plexus and DCP (25), the other one showed no alterations in OCT-A characteristics, yet funduscopic retinal alterations (e.g., cotton wool spots) (26). The data of the present study yielded that especially the intermediate layer (ICP) is affected after COVID-19 infections. Only if COVID-19 disease had shown an even worse clinical progress during SARS-CoV-2 infection were the adjacent retinal microvascular layers affected as well (SVP and DCP). Thus, the present data might explain why the two previous studies showed “contrary” results. Thus, if mild forms of COVID-19 infections would be monitored by OCT-A scans and these devices do not divide the retina into three microvascular layers (the previous studies subdivided into two layers), then mild or moderate affections of microcirculation in the ICP might be masked by the two adjacent unaffected microvascular layers, as the ICP is not scanned by its one. Furthermore, the data of the present study showed a significant impairment of peripapillary VD after COVID-19 disease. To the best of our knowledge, this is the first clinical study on BMO-based APSified peripapillary VD analysis after SARS-CoV-2 infection.

In addition to its diagnostic impact, the results of the present study might be the basis for subsequent analysis of pathophysiological aspects. As VD was reduced significantly even after 138.13 ± 70.67 days, we hypothesize that retinal microcirculation, being a correlate of systemic capillary microcirculation, is affected even long after SARS-CoV-2 infection. As viral SARS-CoV-2 particles were less present in tears and conjunctiva samples (0–7.14%) (45, 46), it might be suggested that clinical retinal findings were triggered by systemic factors during COVID-19 infection. We know that the transition of SARS-CoV-2 from the alveolus to the lung capillaries is crucial for systemic infection and distribution. The endocytosis via ACE-2 receptor is established as the key functional pathway not only for initial infection but also for organ dysfunction (47). However, SARS-CoV-2 is able to cross the border between alveolus and the remaining systems via lung capillaries. A high level of ACE-2 receptor is expressed on endothelial cells being a probable entry to human blood cells. From there, a distribution into any other organs may occur. This pathway is the postulated principal route for the entry into the central nervous system (CNS), leading to neurological symptoms such as dizziness or loss of taste (48). Similarly, COVID-19 enters the liver and may infect hepatocytes, cholangiocytes, and liver endothelium with a high expression of ACE-2 receptor (49). However, infection of endothelial cells is not only an entry way but also an important mediator of organ dysfunction itself. Disruption of the endothelial integrity leads to an activation of the coagulation panel, the downregulation of anti-thrombotic mechanisms (Ang 1-7/MAS 1-R), and the activation of platelets (50). This explains the high risk of thrombosis in large arterial or venous vessels leading to cerebral ischemia (arterial system) or lung emboli. In addition, similar mechanisms of activated coagulation systems have been described in septic shock or systemic inflammatory response syndrome (SIRS). Here, microthrombosis in any capillary system is common. We postulate a similar mechanism due to SARS-CoV-2 in any solid organ with an extensive microvessel system (e.g., retina). Microthrombosis leads to necrotic tissue and deterioration of organ function. This hypothesis is confirmed by the findings of microthrombosis and necrosis in histological studies (51). In addition, alterations of the complete blood count (CBC) might contribute to this pathomechanism. Neutrophilia and lymphopenia, observed in sera of COVID-19 patients, were associated with disease severity (52–55). Going along with an increased number of neutrophils, NET formation occludes the microvessels (immunothrombosis) (21–23, 56). Another important fact is the cross-link between the endothelium and the immune system. Any activation of the coagulation panel leads to a chemotaxis of neutrophils and macrophages with a high cytokine release (57). We observed an overactivation of the immune system in some COVID-19 patients with undulating levels of acute phase proteins and interleukins. Dexamethasone, an overall immunosuppressing drug, shows significant benefit on the prognosis of those patients who no longer suffer from the primary infection but from the secondary problems. Consequently, the exact evaluation of the beginning, quantity, and recovery of micro-vessel thrombosis or rarefication of the capillaries may offer an additional approach to the prediction of organ failure and immunological features such as hemolysis.

A limitation of the study is the very heterogeneous COVID-19-affected population due the disease *per se*, the small number of the cohort, and the relatively young population of controls. Sato et al. could not find a correlation between superficial macular VD and age in the TAIWA study (58). In addition, Park et al. could find no significant difference in parafoveal VD between the age group 20–30 years and age group 40–50 years (59). So we would postulate that the significant difference between the control and post-COVID-19 group was independent of the age difference in the present study, even as the results in the ICP were still significant after age correction of the data. We hypothesize that the severity of capillary impairment after COVID-19 infection is mapped on retinal microcirculation. Thus, the systemic affection of microcirculation might become visible by non-invasive OCT-A technique. As retinal microcirculation is a finely regulated and complex system, analysis of regional alterations of VD might extend the overall VD data. We could observe that the more severe the COVID-19 infection had been, the more alterations in the circular SVP and DCP could be observed, yet not in the overall SVP or overall DCP. These results suggest that next to measurements of overall VD in three retinal micro-vascular layers, regional analysis might increase the diagnostic value of OCT-A in diseases with impaired microcirculation for initial diagnosis and follow-ups.

## Conclusion

Retinal microcirculation may offer a window to the systemic micro-vessel system. We found a remarkable duration of the changed VD in patients who had suffered at COVID-19 infection. The retinal micro-vascular layer in OCT-A imaging (ICP), correlating with the inner nuclear and inner plexiform layers, showed significantly lower microcirculation parameters after SARS-CoV-2 infection compared with healthy eyes, correlating with clinical marker of severity of COVID-19 disease. Future studies regarding the impact of baseline thrombosis prophylaxis might show a clinical impact of these data.

## Data Availability Statement

The original contributions presented in the study are included in the article, further inquiries can be directed to the corresponding authors.

## Ethics Statement

The studies involving human participants were reviewed and approved by Ethics Committee of Erlangen, 295_20B. The patients/participants provided their written informed consent to participate in this study.

## Author Contributions

BH, MG, RS, CM, and MH had the idea. BH, MG, RS, CM, MH, FK, and AB were involved in construction of the study. JH, MM, LR, FH, CS, and MR performed the clinical trial for long-term effects. BH performed the clinical monitoring for short-term effects. LM performed statistical analysis. JF, MM, JH, LR, FH, and CS acquired clinical data. LR, FH, and MR analyzed the OCT-A scans. PL was involved in generation of the draft of the manuscript. BH, CM, MG, MH, RS, MZ, AG, and US-S discussed and interpreted results. BH, MG, and LM were responsible for the draft of the manuscript. All authors contributed to the article and approved the submitted version.

## Conflict of Interest

The authors declare that the research was conducted in the absence of any commercial or financial relationships that could be construed as a potential conflict of interest.
